# Image/Video Coding and Processing Techniques for Intelligent Sensor Nodes

**DOI:** 10.3390/s24154819

**Published:** 2024-07-25

**Authors:** Jinjia Zhou, Ittetsu Taniguchi, Xin Jin

**Affiliations:** 1 Graduate School of Science and Engineering, Hosei University, Tokyo 102-8160, Japan; 2 Graduate School of Information Science and Technology, Osaka University, Osaka 565-0871, Japan; i-tanigu@ist.osaka-u.ac.jp; 3 Shenzhen International Graduate School, Tsinghua University, Shenzhen 518071, China; jin.xin@sz.tsinghua.edu.cn

There is an increasing interest in developing intelligent sensor nodes which enable intelligent processing for Internet of Things (IoT) surveillance, remote sensing, and smart city applications. The data are processed on board by embedded signal processing and machine learning-based analysis algorithms. Such machine learning-driven sensors can transmit key information instead of raw sensing data, lowering the data volume traveling throughout the network.

Due to the explosion of image and video data in IoT systems, specifically designed image and video codecs have been preferred in recent years. With a focus on reducing the data burden and improving the reconstructed image quality, image/video coding and processing techniques performing in low-cost implementations, saving power consumption, and increasing the battery lifetime will cope with the design requirements of sensor nodes. Moreover, intelligent sensors change the traditional intuition-driven sensors in support of machine learning algorithms, delivering high-resolution images and videos for the 5G revolution.

In line with the mission of *Sensors*, the organizers of this Special Issue endeavor to apply the most recent advancements in image/video coding and processing techniques to the development of intelligent sensor nodes. (1) High compression ratio and high reconstruction quality algorithms are presented in this Special Issue. Kiho Choi proposed block partitioning information-based CNN post-filtering for an EVC baseline profile (Contribution 1). Sovann Chen et al. applied a learning-based rate control approach for High Efficiency Video Coding (HEVC) (Contribution 8). (2) Several low-cost video coding hardware designs are introduced. Chen Fu et al. implemented a highly pipelined and highly parallel VLSI architecture of a CABAC encoder for UHDTV applications (Contribution 7). This design can be used for multiple video coding standards such as H.264/AVC, H.265/HEVC, and H.266/VVC. Ionut Schiopu et al. developed a low-complexity lossless coding of asynchronous event sequences for low-power chip integration (Contribution 9). (3) To improve the quality of the compressed video, several techniques are presented in this Special Issue. Zheng Wang et al. introduced standard-dynamic-range to high-dynamic-range television artifact removal techniques by applying a multi-frame Content-Aware Mapping Network (Contribution 2). Zheng Wang proposed an edge-oriented compressed video super resolution that provides users with high-quality and cost-effective HR videos by integrating sensors and codecs (Contribution 4). Wenhao Wan et al. designed a super-resolution video algorithm by adapting single-image super-resolution models (Contribution 6). (4) Several vision-based tasks are presented. Qinyu Wang et al. introduced a transformer-based multiple-object tracking algorithm by applying an anchor-based-query and template matching (Contribution 3). Jun Gong proposed an inpainting algorithm with a separable mask update convolution network (Contribution 5). Dashan Zhang et al. developed a vision-based structural modal identification using hybrid motion magnification (Contribution 10).

In summary, the papers featured in this collection present a variety of innovative approaches and solutions to the challenges in image/video coding and processing techniques for intelligent sensor nodes. The articles introduce algorithms, hardware designs, and deep learning-based strategies to address the development issues of intelligent image sensors. We extend our gratitude to the authors for their contributions and hope that this collection will inspire further advancements in the field of intelligent image/video sensors.

